# Chronic toxicity of low dose monosodium glutamate in albino Wistar rats

**DOI:** 10.1186/s13104-019-4611-7

**Published:** 2019-09-18

**Authors:** Josiah Okwudili Nnadozie, Udunma Olive Chijioke, Okechukwu Charles Okafor, Daniel Bankole Olusina, Angus Nnamdi Oli, Patience Chiebonam Nwonu, Herbert Orji Mbagwu, Chioli Pascal Chijioke

**Affiliations:** 1 Department of Chemical Pathology, Nnamdi Azikiwe University Teaching Hospital, Nnewi, Nigeria; 2 Department of Medical Laboratory Sciences, College Of Medicine, University Of Nigeria Enugu Campus, Enugu, Nigeria; 3 Department of Morbid Anatomy, College Of Medicine, University Of Nigeria Enugu Campus, Enugu, Nigeria; 4 Department of Pharmaceutical Microbiology And Biotechnology, Faculty Of Pharmaceutical Sciences, Nnamdi Azikiwe University, Agulu, Anambra State, Nigeria; 5 Department of Pharmacology & Toxicology, Faculty of Pharmaceutical Sciences, University of Nigeria, Nsukka, Enugu State Nigeria; 6 Department of Pharmacology & Toxicology, College of Medicine, University of Uyo, Uyo, Akwa Ibom State Nigeria; 7 Department of Pharmacology and Therapeutics, College of Medicine, University of Nigeria Enugu Campus, Enugu, Nigeria

**Keywords:** Monosodium glutamate, Chronic toxicity, Albino Wistar rats

## Abstract

**Objective:**

The objective of this study was to observe the effects of chronic dosing with monosodium glutamate on mortality, fertility, major organ functions and histology in albino Wistar rats.

**Results:**

6 male and 6 female rats (age 6 weeks) were bred in a cage, feeding on standard growers’ mash, with monosodium glutamate added (120 mg/kg/day). 12 corresponding breeding rats (on standard feed without MSG) were controls. Chronic dosing with monosodium glutamate in albino Wistar rats (at a dose consistent with the human ADI) led to increased mortality, fertility impairment, and significant changes in major organ function tests and histology. 23 deaths were recorded in the rats fed with MSG additive, while mortality was zero in the control animals. Fertility was lower in rats on MSG (48 births) than in controls (117 births). The weight gain of the MSG rats was higher than in controls. Biochemical parameters and organ histology remained normal in control animals. In MSG-treated rats however, liver/renal function tests, fasting serum cholesterol and triglyceride, serum uric acid showed a significant rise at trimestrial time-points. Histology showed mild portal inflammation in MSG rats, with periglomerular fibrosis and interstitial nephritis in two rats, at 6–12 months.

## Introduction

The non-communicable disease pandemic [1] has been ascribed to modernization [2], in particular the transition from protective Mediterranean [3] or DASH [4] dietary patterns to habitual fast food intake [2, 5].

Apart from sweeteners, salt and hydrogenated fats, the widespread use of monosodium glutamate as a flavour enhancer in modern diets also raises safety concerns, bearing in mind that glutamate is an excitatory neurotransmitter with potential excitotoxicity, mediated by the NMDA receptors [6], as in amyotrophic lateral sclerosis [7] and neuroinflammatory demyelinating disease [8] for example.

Use of MSG has also been linked to nephrotoxicity [9, 10], hepatotoxicity [5, 10], asthma [11] urticaria [12], and neoplastic cell growth and differentiation [13, 14].

Pilot studies in human psoriasis and hypertension suggest that flavour enhancers (including MSG), sweeteners and hydrogenated fats are amongst dietary drivers of adverse gene expression which promote phenotypic expression of the disease (cpcpsoriasis.blogspot.com; [15–18]).

There is need for definitive evidence from controlled studies. Randomized controlled trials in humans have shown a variable role of food additives in causing acute conditions such as angio-oedema and urticaria [19, 20]. However longer controlled trials term in humans (to investigate chronic toxicity of MSG) would be difficult to conduct, as regards compliance with dietary instructions and ethical concerns. Rats are useful models of mammalian excitotoxic neurotransmission [6]. Hence we opted to investigate the subchronic toxicity of MSG in rats, which has been little investigated, apart from reports of urolithiasis and renal damage with chronic MSG intake [9, 21, 22]. Earlier reports on acute toxicity of high dose MSG in rats [10, 23], do not address the issue of chronic toxicity by purportedly safe, low dose MSG in humans. We conducted these chronic toxicity studies using a dosage corresponding to the Acceptable Daily Dietary Intake for humans.

## Main text

### Methods

#### Reagents and materials

Pure Monosodium glutamate, code 101092020, lot no:021M1789V (flavourant) was procured from Sigma Company Germany through their office in Jos. Standard poultry growers’ mash containing 21% protein, 4.4% Fibre, 7.2% fat, 0.5% NaCl and 5.8% ash was procured from Premier Feeds Company Ltd, Enugu. The ELISA kits for the biochemical analyses were products of BioCheck, Inc. S., San Francisco, USA.

#### Care and maintenance of the experimental animals

24 Wistar albino rats of both sexes, aged 8 weeks and weighing 0.1–0.2 kg, were sourced from the Animal house, College of Medicine, University of Nigeria, Enugu Campus, Enugu State. The animals were acclimatized for 2 weeks under natural light–dark cycles. They were given access to clean drinking water and fed ad libitum with standard poultry growers’ mash (specification above). The guidelines as approved by The National Research Council (US) Committee [24] for the care and use of laboratory animals were followed in the maintenance of and experimentation with the animals.The study proposal was approved by the animal ethics committee of the University of Nigeria [UNN/Ethics/001. 03/12/2012].

#### Experimental procedure

The animals were fasted overnight after the acclimatization period and arbitrarily divided into two groups A and B. Group A (6 males and 6 females) served as controls while Group B (also 6 males and 6 females) served as the experimental MSG group. Both groups were fed with similar diets. For Group B monosodium glutamate (MSG) was added to the growers’ mash at a dosage corresponding to the human acceptable daily dietary intake (ADDI, 120 mg/kg) [25]. The animals mated and bred during the study. They were followed up for a year, including veterinary monitoring and advice. Feeding and water intake were monitored to ensure equity amongst the rats and their offspring. Urine excretion was not monitored but droppings and wastes were cleared every morning before providing the standard feed and clean water for the day. Fertility and mortality records were kept for each group. Offspring were kept in separate cages for further observation.

#### Animal sacrifice procedure

Every 3 months, 2 rats were placed in glass jars containing cotton wool soaked with sufficient diethyl ether to induce sleep and anesthesia (as preliminarily ascertained). The glass jar was securely covered for 2 min. Blood samples for biochemistry were collected by ear lobe venepuncture and vital organs were harvested for histology. These procedures are in accordance with guidelines for pain management and humane animal sacrifice [26–29] Biochemical methods used [30–35] were validated with appropriate standards and quality control sera. Statistical methods for data analysis included the Student *t* test, Chi square test, Fisher exact test and analysis of variance with repeated measures (GraphPad PRISM v5.03 for Windows, 2009).

### Results

**Fig. 1:**
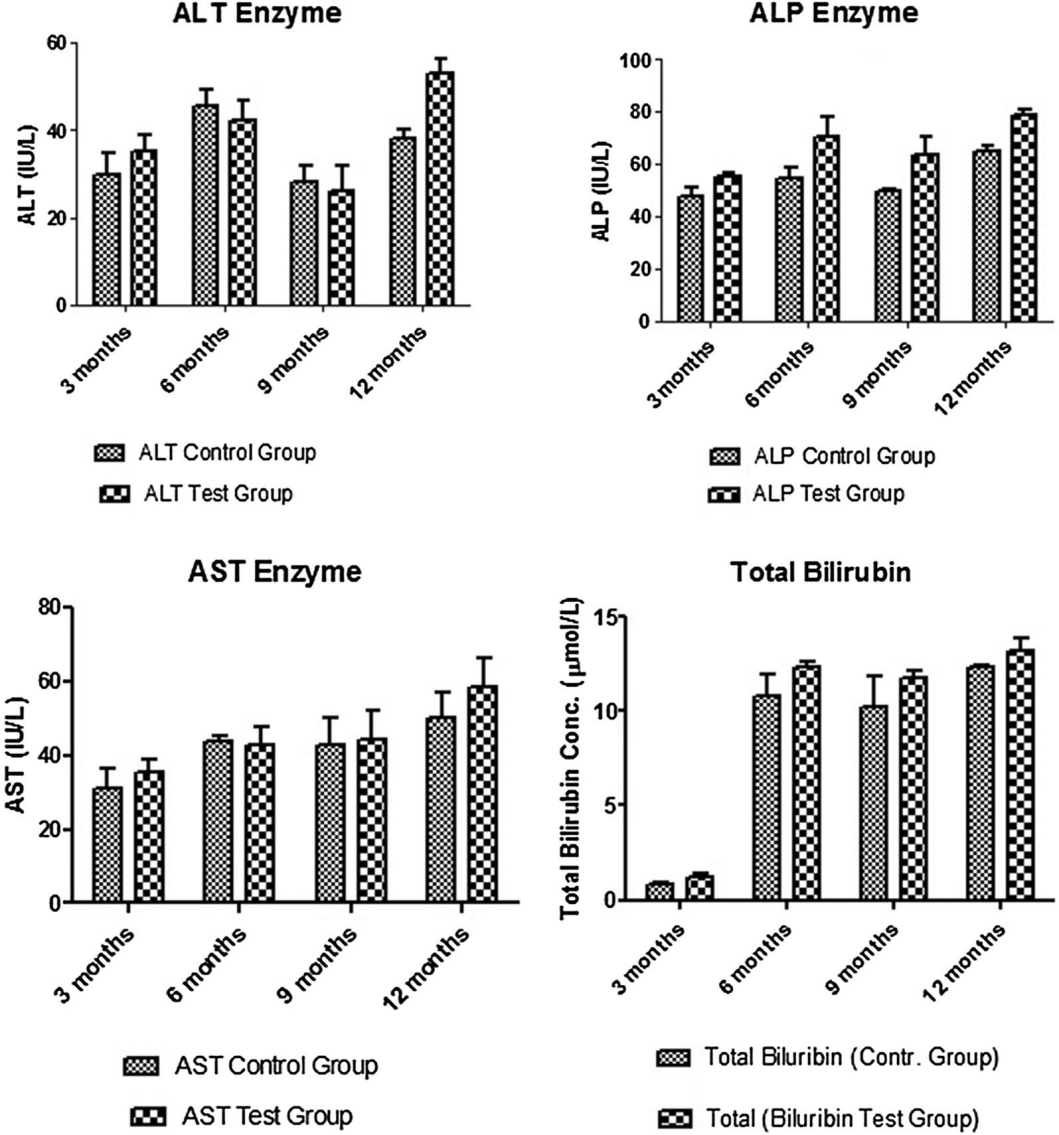
Liver function test results

**Fig. 2:**
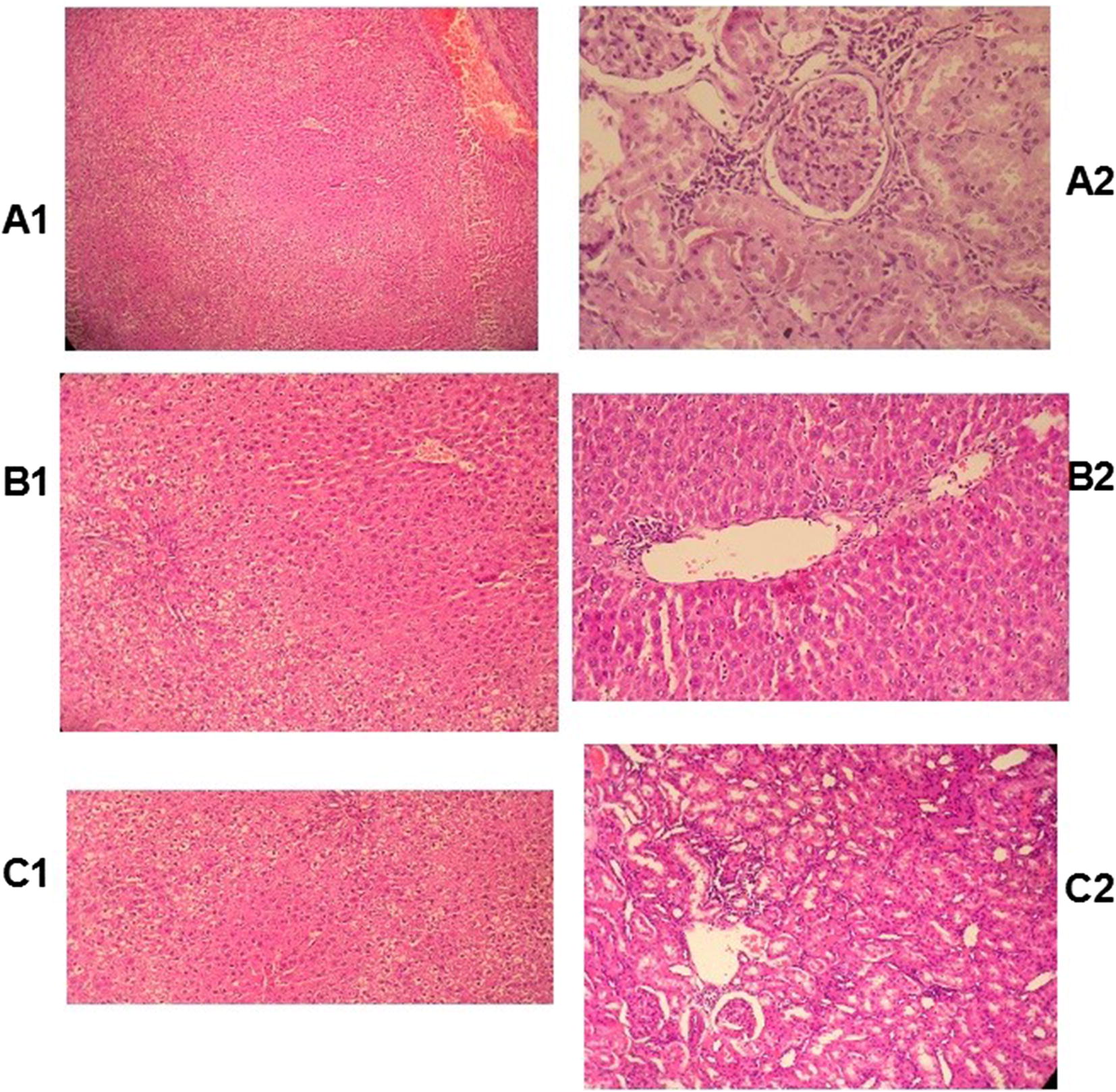
Histology slides comparing control (A2, B2, C2) and MSG (A1, B1, C1) test groups (×100 magnification). The liver showed mild portal inflammation in MSG rats at 9 months and 12 months, with normal histology in controls. Renal histology showed periglomerular fibrosis in one MSG rat at 6 months, and interstitial nephritis in another MSG rat at 12 months

23 deaths were recorded in the MSG rats. There was no mortality in the control animals. Fertility was lower in MSG rats (48 births) than in the controls (117 births). The weight of the MSG rats was consistently higher at the trimestrial time-points (annual mean weight 256 g versus 242 g for controls) (Table 1). Liver function tests showed a consistent rise in the alkaline phosphatase at all time-points, with a rise in the serum transaminases at 3 months and 12 months (Fig. 1). Liver histology showed mild portal inflammation in MSG rats at 9 months and 12 months, with normal histology in controls. While the blood sugar did not show any consistent changes, the fasting lipids showed a rise in the serum cholesterol and triglyceride at 3, 6 and 12 months in MSG rats compared to controls (Additional file 1: Fig. S1). Serum uric acid was increased in MSG rats at 3,6 and 12 months. Serum urea and creatinine were increased in MSG rats at 3, 9 and 12 months (Additional file 2: Fig. S2). Renal histology (Fig. 2) showed periglomerular fibrosis in one MSG rat at 6 months, and interstitial nephritis in another MSG rat at 12 months. Repeated measures ANOVA confirmed statistically significant differences between test and control animals (p < 0.05). There were no consistent differences in serum progesterone and oestradiol concentrations between test and control animals (Additional file 3: Fig. S3). However serum testosterone was significantly lower in the MSG group than in the control group (p = 0.002) (Additional file 3: Fig. S3).

**Table 1:** Mortality, fertility and weight records for the CONTROL and MSG groups

Adult mortality				
	3 months	6 months	9 months	1 year
CONTROL	0	0	0	0
MSG	2	1	0	0

Neonatal mortality				
	3 months	6 months	9 months	1 year
Control	0	3	0	0
MSG	12	8	0	0

Fertility (no. deliveries)				
	3 months	6 months	9 months	1 year
Control	46	50	21	12
MSG	27	28	21	0

	Mean	Weight	(grams)	
	3 months	6 months	9 months	1 year
Control	210	230	250	240
MSG	250	260	265	250

The mean baseline weight for the two groups was 160 g

### Discussion

Chronic dosing with monosodium glutamate in albino Wistar rats (at a dose consistent with the human ADDI) led to fertility impairment, increased adult and neonatal mortality, and significant abnormalities of liver function, renal function, serum lipids, testosterone and uric acid. There were corresponding inflammatory and fibrotic changes seen in the liver and kidney.

The observed abnormalities and inflammatory changes, are recognized in humans to be part of the ‘metabolic syndrome’—associated chronic systemic inflammatory diseases [36]. The epidemiological association of these diseases, suggests a common immunocytopathogenic mechanism [36], as shown for psoriasis and atherosclerosis [37, 38]. The organ specificity of the phenotypic expression of the immune dysfunction would be genetically determined.

Our controlled experimental observations in rats are consistent with an inductive model of the diet-genome interaction in the aetiopathogenesis of chronic diseases, derived from life-long observations on the dietary dependence of phenotypic expression of psoriasis [15, 18]. Strict avoidance of hydrogenated/trans-fats, flavour enhancers (e.g. MSG) and non-sugar sweeteners abates the psoriasis phenotype, with side benefit on associated parameters such as blood pressure, blood sugar, serum lipids and prostate specific antigen [15].

These findings and insights add to concern about the modern dietary trend, and the role that flavour enhancer, sweetener and hydrogenated fat compounds may play in the growing pandemic of immune dysfunction, chronic diseases and male infertility [1, 39]. Contrasting with this trend, for example, are the well reported benefits of traditional Mediterranean dietary habits, which promote longevity and comparatively low chronic disease prevalence and cancer incidence [3].

Glutamate occurs in many foods, such as mushrooms, cured ham, cheese, tomatoes, scallops, tuna, green peas, fish and soy sauces, beef, yeast extract, human and cow’s milk [38]. Adult humans ingest between 10.0 and 20.0 g glutamate per day irrespective of their ethnicity, culinary culture or dietary habits [40].

Daily average consumption estimates for MSG as a food additive include 0.3–1.0 g/day [41] for USA and Europe, 0.6–2.0 g/day in UK [42], 1.5–3.0 g/day in Taiwan, 1.1–1.6 g/day in Japan, 1.6–2.3 g/day in South Korea [43], and 0.56–1.0 g/day in Nigeria [44]. Thus MSG consumption as an additive is about 5–10% of the total daily glutamate intake from various dietary sources [45]. This does not necessarily disculpate MSG from toxicity. For example, ingestion of free unbound glutamate may lead to transiently high or rapidly changing (and hence harmful) plasma concentrations, which do not obtain when glutamate is gradually released from dietary protein and other food sources listed above.

Regulatory authorities approved the safety of MSG as a food additive, based on a lack of convincing evidence in favour of acute or chronic toxicity in animals or humans [46, 47]. Indeed, earlier reports of acute reactions in susceptible individuals e.g. Chinese restaurant syndrome [11] were not convincingly confirmed in randomized trials [19, 20]. More recent reports however, ([5–18, 21–23]: see “Introduction”]) do raise concern, in particular as regards the role that chronic toxicity of MSG may play in engendering the chronic disease process, as exemplified by psoriasis [15, 18] and hypertension [16, 17].

Our experiments emphasize the importance of long term food and drug safety monitoring. The conventional safety monitoring undertaken in preclinical and phase one clinical trials tends to focus on acute and subacute toxicity monitoring, with little regard for the possibility of chronic toxicities. The need for ‘phase six’ clinical trials has recently been suggested. [40]. These involve withdrawal from potentially toxic long term drug exposures. Correspondingly, there is a need for clinical trials of withdrawal from long-term dietary exposures, including flavour enhancer and sweetener exposures, which putatively promote adverse gene expression and hence chronic disease phenotypes.

Further work should include rat breeding studies to confirm the suspected nutritoxigenetic effects, since the human observational studies point towards genetically predisposed toxicity of MSG. It should be possible to breed rat strains which show increased susceptibility to MSG toxicities, helping to buttress the intuitive hypothesis as regards the incubation of chronic diseases and cancers by the modern dietary trend. Pinpointing the likely culprit dietary components would allow the design of human clinical trials and community intervention programmes to address the burgeoning pandemics of immune dysfunction, chronic diseases and cancers.

## Limitations

- These novel data on chronic toxicity of monosodium glutamate in rats cannot necessarily be extrapolated to humans. However they suggest the need for appropriate clinical trials of MSG avoidance in humans.
- The lack of research funding restricted the number of rats to only 24 (12 experimental animals and 12 controls). This limits the statistical significance of results. Studies in larger numbers of rats need to be conducted.
- Logistic difficulties prevented prompt post mortem examinations on adult and neonatal rats which died. Hence striking toxic effects of MSG which caused the mortality may have been missed. This should be investigated in further studies.
- MSG is only one of various modern diet ingredients found by the last author to engender and aggravate psoriasis, a prototypical chronic disease. Chronic toxicity studies should also be conducted on other postulated egregious ingredients (e.g. artificial sweeteners, emulsifiers, trans-fats, hydrogenated fats), alone and combined. This would further guide personalized food avoidance clinical trials to address the waxing pandemic of non-communicable diseases.

## Supplementary information

**Additional file** **1****: Fig. S1.** Serum lipids and glucose concentrations.

**Additional file** **2****: Fig. S2.** Serum urea, creatinine and uric acid concentrations.

**Additional file** **3****: Fig. S3.** Sex hormone concentrations.

## Data Availability

The datasets used and/or analysed during the current study are available from the corresponding author on reasonable request.

## References

[1] World Health Organization (2014). Global status report on noncommunicable diseases.

[2] Mckeown A (1983). Classification of disease: a basis for health strategies. Br Med J.

[3] Simopoulos AP (2001). The Mediterranean diets: what is so special about the diet of Greece? The scientific evidence. J Nutr.

[4] Parikh A, Lipsitz SR, Natarajan S (2009). Association between a DASH-like diet and mortality in adults with hypertension: findings from a population based follow up study. Am J Hypertens.

[5] Kechagias S, Ermersson A, Dahlqvist O, Lundberg P, Lindstrom T (2008). Fast food based hyper-alimentation can induce rapid and profound elevation of serum alanine aminotransferase in healthy subjects. Gut.

[6] Rothman SM, Olney JW (1995). Excitotoxicity and the NMDA receptor: still lethal after eight years. Trends Neurosci.

[7] Pandya RS, Zhu H, Li W, Bowser R, Friedlander RM, Wang X (2013). Therapeutic neuroprotective agents for amyotrophic lateral sclerosis. Cell Mol Life Sci.

[8] Bolton C, Paul C (2006). Glutamate receptors in neuroinflammatory demyelinating disease. Mediat Inflamm.

[9] Sharma A (2015). Monosodium glutamate induced oxidative kidney damage and possible mechanisms: a mini-review. J Biomed Sci.

[10] Ortiz GG, Bitzer Quintero OK, Zarate CB, Rodriguez Reynoso S, Larios Arceo F (2006). Monosodium glutamate induced damage in liver and kidney: a morphological and biochemical approach. Biomed Pharmacother.

[11] Allen DH, Baker G (1981). Chinese restaurant asthma. New Eng J Med.

[12] Botey J, Cozzo M, Marin A, Eseverri JL (1988). Monosodium glutamate and skin pathology in pediatric allergology. Allergol Immunopathol.

[13] Viallard V, Denis C, Trocheris V, Murat JC (1986). Effect of glutamine deprivation and glutamate or ammonium chloride addition on growth rate, metabolism and differentiation of human colon cancer cell line HT29. Int J Biochem.

[14] Zhang Y, Lu X, Bhavnani BR (2003). Equine estrogens differentially inhibit DNA fragmentation induced by glutamate in neuronal cells by modulation of regulatory proteins involved in programmed cell death. BMC Neurosci.

[15] Chijioke CP, Chijioke OU, Okolo T (2012). Personalized diet for psoriasis: side benefit on blood pressure and metabolic parameters. J Hypertens.

[16] Chijioke C, Chijioke O, Okolo T, Ekwe E, Nubila I, Onwasigwe C (2014). Nutritoxigenetics of hypertension: efficacy of a personalized categorical food avoidance dietary approach. Basic Clin Pharmacol Toxicol.

[17] Chijioke CP, Chijioke UO, Okolo T, Ekwe E, Onwasigwe C (2014). Case study of essential hypertension: efficacy of an antipsoriatic dietary approach. J Hypertens.

[18] Chijioke CP, Chijioke UO, Okolo T, Ekwe E (2014). Nutritoxigenetics of psoriasis: a ten year online case diary of dietary challenge and avoidance effects on chronic disease phenotype. Basic Clin Pharmacol Toxicol.

[19] Yang WH, Drouin MA, Herbert M, Mao Y, Karsh J (1997). The monosodium glutamate symptom complex: assessment in a double blind placebo controlled randomized study. J Allergy Clin Immunol.

[20] Geha RS, Beiser A, Ren C, Patterson R, Greenberger PA (2000). Review of alleged reaction to monosodium glutamate and outcome of a multicenter double blind placebo controlled study. J Nutr.

[21] Sharma A, Prasongwattana V, Cha’on U, Selmi C, Hipkaeo W, Boonnate P (2013). Monosodium glutamate consumption is associated with urolithiasis and urinary tract obstruction in rats. PLoS ONE.

[22] Sharma A, Wongkham C, Prasongwattana V, Boonnate P, Thanan R, Reungjui S (2014). Proteomic analysis of kidney in rats chronically exposed to monosodium glutamate. PLoS ONE..

[23] Eweka AO (2007). Histological studies of the effects of monosodium glutamate on the kidney of adult Wistar rats. Internet J Health.

[24] National Research Council (US) Committee for the Update of the Guide for the Care and Use of Laboratory Animals. Guide for the Care and Use of Laboratory Animals. 8th edition. Washington (DC): National Academies Press (US); 2011. https://www.ncbi.nlm.nih.gov/books/NBK54050/10.17226/12910. 21595115

[25] Joint FAO/WHO expert committee on food additives (2006). food additives. Toxicol Sci.

[26] Animals (Scientific Procedures) Act 1986 (as amended). Home Office (UK). 2013.

[27] Canadian Council on Animal Care (2010). Training module on pain, distress and end point.

[28] Veterinary Surgeon Act Cap V3 LFN 2004, Federal Republic of Nigeria. “The Care and Use of Animals for Scientific Purposes”.

[29] Animal Diseases (Control) Act. Cap A17 LFN, 2004, Federal Republic of Nigeria “The Care and Use of Animals for Scientific Purposes”.

[30] Reitman S, Frankel S (1957). Alkaline phosphatase estimation. Am J Clin Pathol.

[31] Tietz N (2006). Clinical guide to laboratory tests.

[32] Fawiett JK, Scott JE (1960). Urease method of analysis. J Clin Pathol.

[33] Allain P, Chan C, Richond W, Fu P (1974). Enzymatic determination of total serum cholesterol. Clin Chem.

[34] Trinder P (1969). Determination of blood glucose using an oxidase peroxidase system with non-carcinogenic chromogen. J Clin Pathol.

[35] Carl A, Edward RA, David EB (2010). Uric acid. Tietz fundamentals of clinical chemistry.

[36] Mrowietz U, Elder JT, Baker J (2006). The importance of disease associations and concomitant therapy for the long term management of psoriasis patients. Dermatol Res.

[37] Späh F (2008). Inflammation in atherosclerosis and psoriasis: common pathogenic mechanisms and the potential for an integrated treatment approach. Br J Dermatol.

[38] Yamaguchi S, Ninomiya K (2000). Umami and food palatability. J Nutr.

[39] Andersson AM, Jørgensen N, Main KM, Toppari J, Rajpert-De Meyts E, Leffers H, Juul A, Jensen TK, Skakkebaek NE (2008). Adverse trends in male reproductive health: we may have reached a crucial ‘tipping point’. Int J Androl.

[40] Fernstrom JD (1994). Dietary amino acids and brain function. J Am Diet Assoc.

[41] Beyreuther K, Biesalski HK, Fernstrom JD, Grimm P, Hammes WP, Heinemann U, Kempski O, Stehle P, Steinhart H, Walker R (2007). Consensus meeting: monosodium glutamate—an update. Eur J Clin Nutr.

[42] Rhodes J, Titherley AC, Norman JA, Wood R, Lord DW (1991). A survey of the monosodium glutamate content of foods and an estimation of the dietary intake of monosodium glutamate. Food Addit Contam.

[43] Lee EH, Lee DI (1986). A study of intake of monosodium glutamate in Korea. Korean J. Environ. Healtth Soc.

[44] Unaeze HN (2010). Consumer knowledge attitude and practice towards theuse of monosodium glutamate and food grade bouillon cubes as dietary constituents. Pak J Nutr.

[45] Brosnan JT, Drewnowski A, Friedman MI (2014). Is there a relationship between dietary MSG and obesity in animals?. Amino Acids.

[46] Walker R, Lupien JR (2000). J Nutr.

[47] Maluly HDB, Arisseto-Bragotto AP, Reyes FGR (2017). Monosodium glutamate as a tool to reduce sodium in foodstuffs: technological and safety aspects. Food Sci Nutr.

