# Correction to: Chronic toxicity of low dose monosodium glutamate in albino Wistar rats

**DOI:** 10.1186/s13104-020-4888-6

**Published:** 2020-01-09

**Authors:** Josiah Okwudili Nnadozie, Udunma Olive Chijioke, Okechukwu Charles Okafor, Daniel Bankole Olusina, Angus Nnamdi Oli, Patience Chiebonam Nwonu, Herbert Orji Mbagwu, Chioli Pascal Chijioke

**Affiliations:** 1 Department of Chemical Pathology, Nnamdi Azikiwe University Teaching Hospital, Nnewi, Nigeria; 2 Department of Medical Laboratory Sciences, College of Medicine, University of Nigeria Enugu Campus, Enugu, Nigeria; 3 Department of Morbid Anatomy, College of Medicine, University of Nigeria Enugu Campus, Enugu, Nigeria; 4 Department of Pharmaceutical Microbiology and Biotechnology, Faculty of Pharmaceutical Sciences, Nnamdi Azikiwe University, Agulu, Anambra State, Nigeria; 5 Department of Pharmacology & Toxicology, Faculty of Pharmaceutical Sciences, University of Nigeria, Nsukka, Enugu State Nigeria; 6 Department of Pharmacology & Toxicology, College of Medicine, University of Uyo, Uyo, Akwa Ibom State Nigeria; 7 Department of Pharmacology and Therapeutics, College of Medicine, University of Nigeria Enugu Campus, Enugu, Nigeria

## Correction to: BMC Res Notes (2019) 12:593 10.1186/s13104-019-4611-7

The original article [1] mistakenly omits mention of grant funding which partially funded the work undertaken in this article. The authors sincerely apologise for this omission and would like to acknowledge this funding source in this correction article as per the below information:

1. TETFund (Tertiary Education Trust Fund): Institution based TETFund grant. [TETFUND/DESS/UNI/NSUKKA/2017/RP/VOL.I].
