# Supplementary figures and images for: Chronic toxicity of low dose monosodium glutamate in albino Wistar rats
Source: BMC Res Notes. 2019 Sep 18;12:593. doi: 10.1186/s13104-019-4611-7 (PMC6751858; doi:10.1186/s13104-019-4611-7)

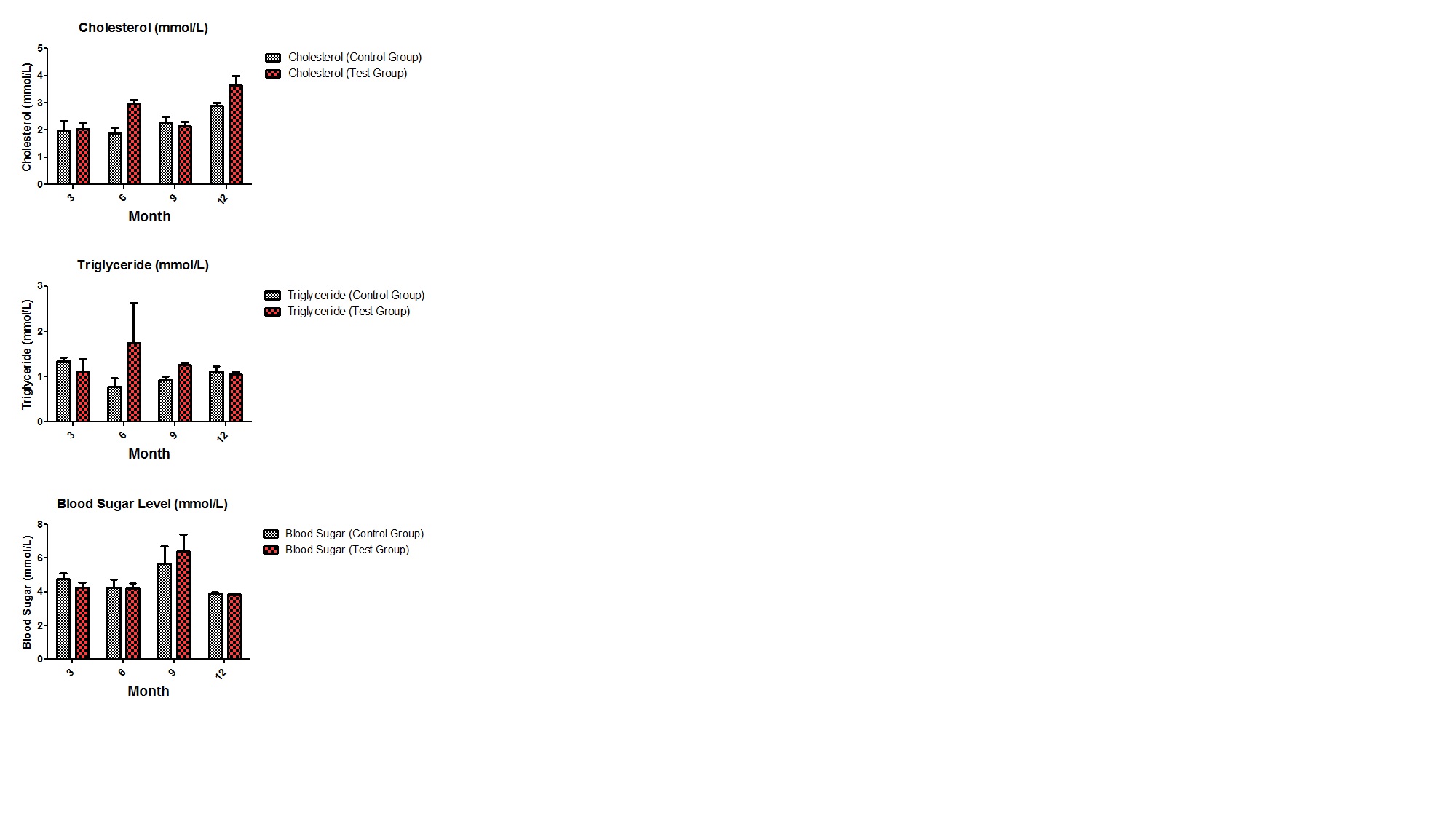

Supplement: Supplementary file 1 — Additional file 1: Fig. S1. Serum lipids and glucose concentrations. [file 13104_2019_4611_MOESM1_ESM.jpg]

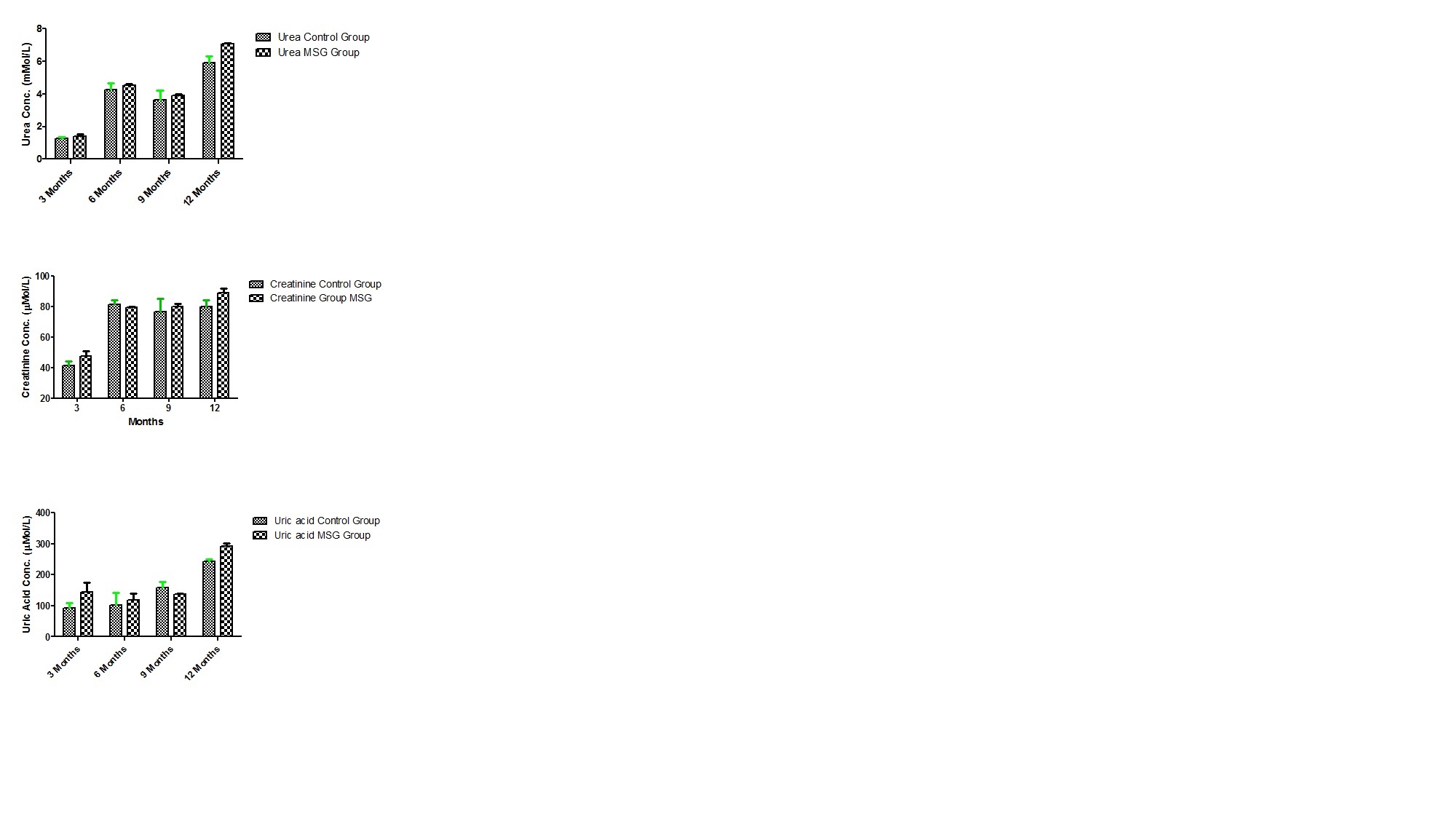

Supplement: Supplementary file 2 — Additional file 2: Fig. S2. Serum urea, creatinine and uric acid concentrations. [file 13104_2019_4611_MOESM2_ESM.jpg]

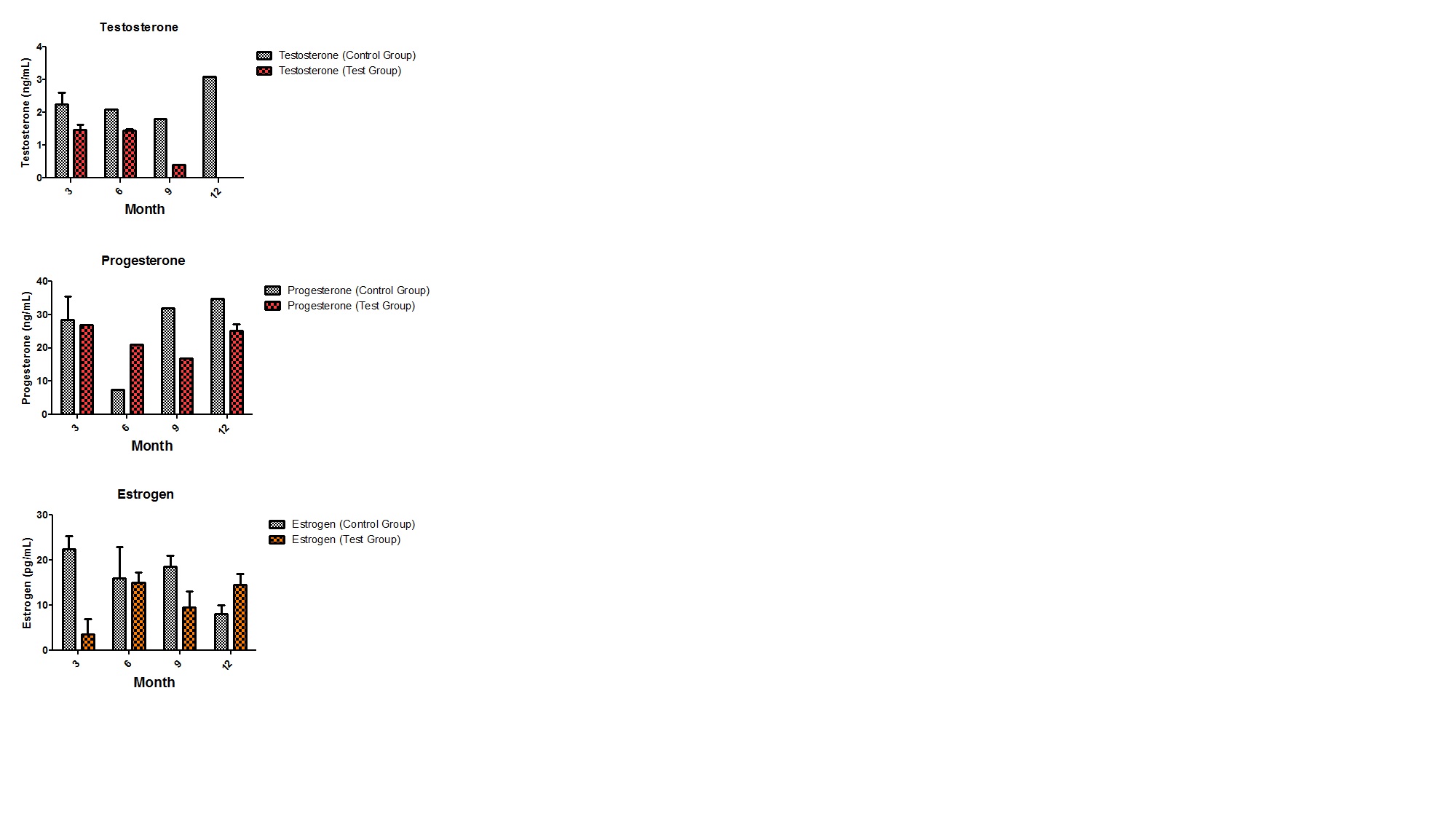

Supplement: Supplementary file 3 — Additional file 3: Fig. S3. Sex hormone concentrations. [file 13104_2019_4611_MOESM3_ESM.jpg]
